# Implementation research on kangaroo mother care, Bangladesh

**DOI:** 10.2471/BLT.20.284158

**Published:** 2021-10-26

**Authors:** ANM Ehtesham Kabir, Sharmin Afroze, Zubair Amin, Agnihotri Biswas, Sabina Ashrafee Lipi, Mahbuba Khan, Khaleda Islam, Shamsul Haque, MAK Azad Choudhury, Mohammod Shahidullah

**Affiliations:** a National Newborn and Child Health Cell, Directorate General of Health Services, Dhaka, Bangladesh.; b Dr MR Khan Shishu Hospital and Institute of Child Health, Dhaka, Bangladesh.; c Khoo-Teck Puat-National University Children’s Medical Institute, National University Health System (NUHS), Tower Block, Level 12, 1E Kent Ridge Road, Singapore 119228, Singapore.; d National Newborn Health Program, Directorate General of Health Services, Dhaka, Bangladesh.; e World Health Organization, Dhaka, Bangladesh.; f Maternal Neonatal Child and Adolescent Health, Directorate General of Health Services, Dhaka, Bangladesh.; g Dhaka Shishu (Children) Hospital, Dhaka, Bangladesh.; h Bangabandhu Sheikh Mujib Medical University, Dhaka, Bangladesh.

## Abstract

**Objective:**

To describe the implementation, coverage and performance of the national kangaroo mother care programme in Bangladesh.

**Methods:**

Kangaroo mother care services for clinically stable babies with birth weight under 2000 g were set up in government-run health-care facilities in rural and urban areas of Bangladesh. Each facility provided counselling on kangaroo mother care, ensured adequate nutrition, and followed up mothers and babies. We studied implementation of the programme from January 2016 to March 2020 using data from the national database. We tracked the number of eligible babies enrolled and their outcomes, mortality and post-discharge follow-up.

**Findings:**

The numbers of kangaroo mother care facilities increased from 16 in 2016 to 108 in 2020. Over the 4-year period 64 426 babies weighing under 2000 g were born in these facilities,  6410 of whom received kangaroo mother care. The quarterly percentage of eligible babies receiving kangaroo mother care increased from 4.7% (37/792) during the first quarter to 21.7% (917/4226) during the last five quarters of the programme. Deaths of babies receiving kangaroo mother care showed a downward trend over the study period. The overall mortality was 1.2% (77/6410), with large quarterly fluctuations in mortality. Post-discharge follow-up was low and only 15–20% of babies received four follow-up visits.

**Conclusion:**

Implementation of kangaroo mother care interventions is feasible in low-resource settings. Such care has the potential to reduce mortality among low-birth-weight and premature babies. Challenges include low coverage, expanding the programme to the community and strengthening the monitoring system.

## Résumé

**Objectif:**

Décrire la mise en œuvre, la couverture et la performance du programme national de la méthode kangourou au Bangladesh.

**Méthodes:**

Des services de soins maternels appliquant la méthode kangourou pour les bébés cliniquement stables d’un poids de naissance inférieur à 2000 g ont été mis en place dans des établissements de santé publics des zones rurales et urbaines du Bangladesh. Chaque établissement a fourni des conseils sur la méthode kangourou, a veillé à une nutrition adéquate et a suivi les mères et les bébés. Nous avons étudié la mise en œuvre de ce programme de janvier 2016 à mars 2020 en utilisant les données de la base de données nationale. Nous avons suivi le nombre de bébés éligibles inscrits et leurs résultats, la mortalité et la prise en charge après la sortie de l’hôpital.

**Résultats:**

Le nombre d’établissements de soins de santé appliquant la méthode kangourou a augmenté, passant de 16 en 2016 à 108 en 2020. Sur cette période de 4 ans, 64 426 bébés pesant moins de 2000 g sont nés dans ces établissements, dont 6410 ont reçu des soins selon la méthode kangourou. Le pourcentage trimestriel de bébés éligibles recevant des soins selon la méthode kangourou a augmenté de 4,7 % (37/792) au cours du premier trimestre à 21,7% (917/4226) au cours des cinq derniers trimestres du programme. Le taux de décès de bébés bénéficiant de la méthode kangourou a montré une tendance à la baisse pendant la période d’étude. La mortalité globale était de 1,2% (77/6410), avec de fortes fluctuations trimestrielles de la mortalité. La prise en charge après la sortie de l’hôpital était faible, et seuls 15 à 20 % des bébés ont bénéficié de quatre visites de suivi.

**Conclusion:**

La mise en œuvre d’interventions fondées sur la méthode kangourou est réalisable dans des contextes à faibles ressources. Cette méthode a le potentiel de réduire la mortalité des prématurés et des bébés dont le poids à la naissance est faible. Les défis à relever comprennent une faible couverture, l’extension du programme à la communauté et le renforcement du système de suivi.

## Resumen

**Objetivo:**

Describir la implementación, la cobertura y el rendimiento del programa nacional sobre el método madre canguro en Bangladesh.

**Métodos:**

Se crearon servicios que ofrecen el método madre canguro para bebés estables en términos clínicos y con un peso inferior a 2000 g al nacer, en centros sanitarios públicos de zonas rurales y urbanas de Bangladesh. Cada centro proporcionó asesoramiento sobre el método madre canguro, garantizó una nutrición adecuada y realizó un seguimiento de las madres y los bebés. Se estudió la implementación del programa desde enero de 2016 hasta marzo de 2020 utilizando los datos de la base de datos nacional. Se hizo un seguimiento del número de bebés idóneos inscritos y de sus resultados, de la mortalidad y del seguimiento posterior al alta.

**Resultados:**

El número de centros que ofrecen el método madre canguro aumentó de 16 en 2016 a 108 en 2020. A lo largo del periodo de 4 años nacieron 64 426 bebés en estos centros, con un peso inferior a 2000 g, de los que 6410 recibieron atención de madre canguro. El porcentaje trimestral de bebés idóneos que recibieron atención de madre canguro aumentó del 4,7 % (37/792) durante el primer trimestre al 21,7 % (917/4226) durante los últimos cinco trimestres del programa. Las muertes de los bebés que recibieron atención de madre canguro mostraron una tendencia a la baja a lo largo del periodo de estudio. La mortalidad global fue del 1,2 % (77/6410), con grandes fluctuaciones trimestrales en la mortalidad. El seguimiento posterior al alta fue bajo y solo entre el 15 y el 20 % de los bebés recibieron cuatro consultas de revisión.

**Conclusión:**

La implementación de las intervenciones del método madre canguro es factible en entornos de bajos recursos. Este tipo de atención tiene el potencial de reducir la mortalidad entre los bebés de bajo peso al nacer y los prematuros. Los problemas son la baja cobertura, la ampliación del programa a la comunidad y el fortalecimiento del sistema de control.

## ملخص

**الغرض:**

وصف كل من تنفيذ، وتغطية، وأداء البرنامج الوطني لنظام رعاية الأم لوليدها على طريقة الكنغر في بنغلاديش.

**الطريقة:**

تم تأسيس خدمات نظام رعاية الأم لوليدها على طريقة الكنغر للمواليد المستقرين سريريًا، والذين يقل وزنهم عند الولادة عن 2000 جرام، في مرافق الرعاية الصحية التي تديرها الحكومة في المناطق الريفية والحضرية في بنغلاديش. قام كل مرفق بتقديم المشورة بشأن نظام رعاية الأم لوليدها على طريقة الكنغر، مع ضمان توفير التغذية المناسبة، ومتابعة الأمهات والمواليد. درسنا تنفيذ البرنامج من يناير/كانون ثاني 2016 إلى مارس/آذار 2020 باستخدام بيانات من قاعدة البيانات الوطنية. كما قمنا بتتبع عدد الأطفال المؤهلين المسجلين، ونتائجهم، والوفيات، ومتابعة ما بعد الخروج من المرافق.

**النتائج:**

ارتفع عدد المرافق العاملة بنظام رعاية الأم لوليدها على طريقة الكنغر من 16 في عام 2016 إلى 108 في عام 2020. وعلى مدار فترة 4 سنوات، تمت ولادة 64426 طفلًا يزنون أقل من 2000 جرام في هذه المرافق، تلقى 6410 منهم رعاية الأم لوليدها على طريقة الكنغر. ارتفعت النسبة الربعية للمواليد المؤهلين، الذين يتلقون رعاية الأم لوليدها على طريقة الكنغر، من 4.7% (37/792) خلال الربع الأول إلى 21.7% (917/4226) خلال الأرباع الخمسة الأخيرة من البرنامج. أظهرت وفيات الأطفال الذين يتلقون رعاية الأم لوليدها على طريقة الكنغر، اتجاهاً تنازلياً خلال فترة الدراسة. كان إجمالي معدل الوفيات 1.2% (77/6410)، مع تقلبات ربع سنوية كبيرة في معدل الوفيات. كانت متابعة ما بعد الخروج من المرافق منخفضة، وتلقى 15 إلى 20% فقط من الأطفال أربع زيارات متابعة.

**الاستنتاج:**

يُعد تنفيذ تدخلات رعاية الأم لوليدها على طريقة الكنغر أمرًا ممكنًا في ظل أوضاع انخفاض الموارد. مثل هذه الرعاية لديها القدرة على الحد من الوفيات بين الأطفال الذين يعانون من انخفاض الوزن عند الولادة وأطفال الولادة المبكرة. وتشمل التحديات انخفاض التغطية، وتوسيع البرنامج للمجتمع، وتدعيم نظام الرصد.

## 摘要

**目的:**

描述孟加拉国在全国范围内采用袋鼠式护理计划的实施、覆盖范围和效果。

**方法:**

在孟加拉国城市和乡村地区政府运营的医疗机构中为出生体重低于 2000 克的临床表现稳定的婴儿提供袋鼠式护理服务。每个医疗机构都提供袋鼠式护理咨询服务，确保提供充足的营养，以及对母婴状况进行跟踪随访。我们使用国家数据库中的数据，研究了 2016 年 1 月至 2020 年 3 月期间该计划的实施情况。我们跟踪了一定数量符合条件的登记婴儿和他们接受袋鼠式护理后的表现、死亡率以及出院随访情况。

**结果:**

实施袋鼠式护理的医疗机构数量从 2016 年的 16 个增加到 2020 年的 108 个。在 4 年期间，这些机构中有 64,426 名体重低于 2000 克的婴儿出生，其中 6,410 名婴儿接受了袋鼠式护理。在该计划的最后五个季度，合格婴儿接受袋鼠式护理的季度百分比从第一季度的 4.7% (37/792) 增加到 21.7% (917/4226)。在研究期间，接受袋鼠式护理的婴儿死亡率呈下降趋势。总死亡率为 1.2% (77/6410)，死亡率每季度浮动较大。出院后接受随访的婴儿不多，仅有 15-20% 的婴儿接受了四次随访。

**结论:**

在资源匮乏的情况下实施袋鼠式护理干预是可行的。该护理方式有可能减少出生体重过低的婴儿和早产婴儿的死亡率。但仍面临许多挑战，包括覆盖范围较低，将计划推行至社区，以及加强监测系统。

## Резюме

**Цель:**

Описать внедрение, охват и эффективность национальной программы по внедрению метода кенгуру в Бангладеш.

**Методы:**

В государственных медицинских учреждениях в сельских и городских районах Бангладеш были созданы условия для внедрения метода кенгуру для клинически стабильных детей с массой тела при рождении менее 2000 г. В каждом учреждении проводились консультации относительно метода кенгуру, обеспечивалось надлежащее питание и проводилось наблюдение за матерями и детьми. Авторы изучили внедрение программы с января 2016 года по март 2020 года с использованием данных из национальной базы данных. Авторы наблюдали за детьми, соответствующими критериям отбора, за их исходами, смертностью и результатами наблюдения после выписки.

**Результаты:**

Количество учреждений, использующих метод кенгуру, увеличилось с 16 в 2016 году до 108 в 2020 году. За 4 года в этих учреждениях родилось 64 426 младенцев весом до 2000 г. Из них для 6410 младенцев использовался метод кенгуру. Ежеквартальный процент детей, соответствующих критериям отбора для получения метода кенгуру, увеличился с 4,7% (37/792) в первом квартале до 21,7% (917/4226) в течение последних пяти кварталов программы. Смертность среди младенцев, получавших метод кенгуру, имела тенденцию к снижению на протяжении периода исследования. Общая смертность составила 1,2% (77/6410) со значительными ежеквартальными колебаниями смертности. Последующее наблюдение проводилось на низком уровне, и только 15–20% младенцев были посещены 4 раза после выписки.

**Вывод:**

Внедрение метода кенгуру является целесообразным в странах с низким уровнем материальных ресурсов. Такой метод может снизить смертность среди детей с низкой массой тела при рождении и недоношенных детей. Задачи, которые предстоит решить: низкая степень охвата, расширение программы среди населения и укрепление системы мониторинга.

## Introduction

Prematurity and its complications are one of the leading causes of neonatal deaths worldwide. Every year 15 million babies in the world are born prematurely.^1^ Low birth weight, defined as babies born with birth weight below 2500 g, frequently coexists with prematurity. Lower birth weight is associated with higher mortality, especially below birth weight 2000 g.^2^

Kangaroo mother care is a low-cost, low-technology intervention, defined as “a standard, protocol-based care system for preterm and/or low birth-weight newborns based on skin-to-skin contact between the newborn and the mother or the caregiver.”^3^ More than 200 hospital-based studies have compared incubator care with kangaroo mother care and determined that kangaroo mother care is effective in maintaining adequate temperature, reducing hospital-acquired infections, encouraging exclusive breastfeeding and promoting neonatal weight gain.^3^ Estimates show that kangaroo mother care could prevent up to 450 000 preterm deaths each year if near universal coverage of the practice is achieved.^4^ Although success of kangaroo mother care has been widely reported from hospital-based interventions,^5^ there are fewer published studies of the implementation of kangaroo mother care at a national level.

Bangladesh, a lower-middle-income country, has made significant progress in reducing child mortality. However, neonatal mortality is still high and the burden of prematurity is increasing. Bangladesh is one of the top 10 countries with the largest number of preterm births.^6^ An estimated 31% of all neonatal deaths in Bangladesh are due to prematurity and its complications.^7^ The World Health Organization (WHO) recommends kangaroo mother care for routine care of newborns weighing below 2000 g at birth to be initiated in health-care facilities as soon as the newborn is clinically stable.

In 2013, the Government of Bangladesh declared its commitment to introduce and scale up kangaroo mother care in health-care facilities, with continuation of care at home. To end preventable child deaths by 2035, the government renewed its declaration in *Child survival call to action: a promise renewed*.^8^^,^^9^ One of the aims was to reduce the under-five mortality rate to 20 per 1000 live births by 2035 through implementation of different strategies including kangaroo mother care for low-birth-weight babies.^8^ A technical subgroup was formed under the guidance of Bangladesh’s national technical working committee for newborn health. In 2014, the committee developed the national guideline on kangaroo mother care in line with the WHO kangaroo mother care guideline.^10^ The national guideline was designed for health-care providers and managers and focused on the introduction, expansion and strengthening of kangaroo mother care practices. In 2015, kangaroo mother care was included as one of the key strategies in the Bangladesh Every Newborn Action Plan.^11^ The main activities planned were: (i) ensuring counselling on kangaroo mother care in health-care facilities; (ii) providing follow-up kangaroo mother care services in the community; (iii) establishing centres of excellence for kangaroo mother care in secondary and tertiary level facilities; and (iv) setting targets for initiation of kangaroo mother care. In 2016, kangaroo mother care was incorporated as a priority newborn health intervention in the implementation plan of the 4th Health, Population and Nutrition Sector Programme of the Ministry of Health and Family Welfare.^12^

We describe the first 4 years of implementation of the kangaroo mother care programme in Bangladesh. We aimed to demonstrate that implementation of the programme in a low-resource country is feasible and can reduce neonatal mortality. We also compiled lessons learnt to inform further scale-up of the programme in Bangladesh and other low- and middle-income countries with a similar socioeconomic context.

## Methods

### Setting

The government introduced kangaroo mother care nationwide as a part of its comprehensive strategy to reduce neonatal mortality.^12^^,^^13^ The target was to establish kangaroo mother care at 20% of public health facilities by 2016 and at 50% of public health facilities by 2020.^9^ Kangaroo mother care services were planned for subdistrict health complexes and higher-level facilities (district hospitals and medical college hospitals) in both urban and rural areas. Under the programme, a dedicated space (corner) for kangaroo mother care was established in each designated health facility. Although 142 kangaroo mother care corners were originally planned, by 2020 only 108 corners remained functional under the supervision of the Directorate General of Health Services. In addition, 22 kangaroo mother care corners were established under the Directorate General of Family Planning, but we only evaluated kangaroo mother care facilities under the Directorate General of Health Services.

### Implementation

In 2014–2015, as a part of the implementation plan for kangaroo mother care, the government developed a national trainer pool for health-care professionals. Training programmes included a 3-day training on kangaroo mother care focusing on practical demonstration, learning by doing and facility visits for direct observation of kangaroo mother care services. The programme also included staff training on recording and reporting of data. The national newborn health programme implemented a social and behavioural change communication campaign to create awareness in the community via printed materials (such as leaflets, posters, flipcharts and booklets), television, radio and social media platforms. 

**Fig. 1:**
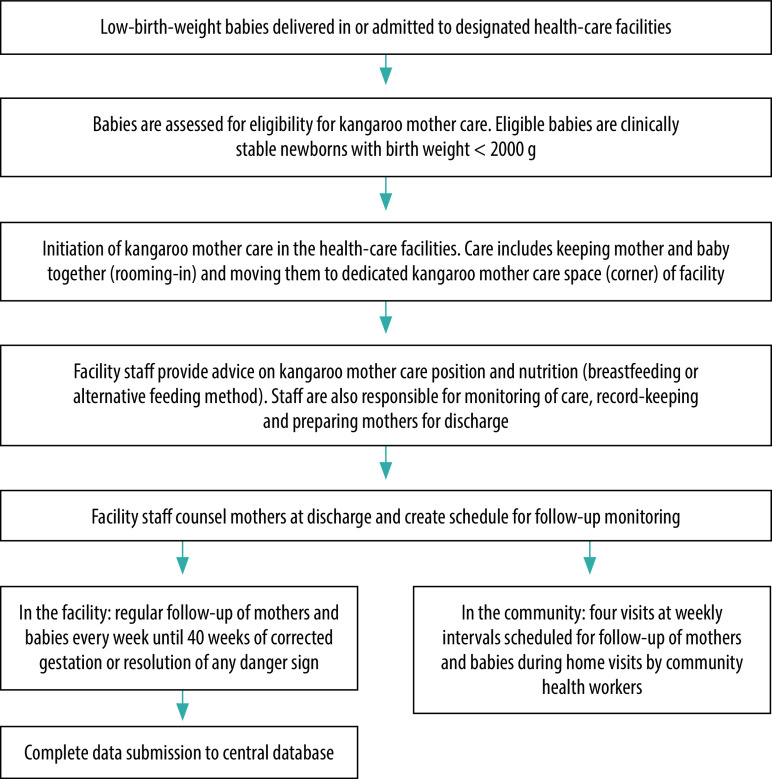
Pathway of care for babies born in designated kangaroo mother care centres, Bangladesh

In 2016, kangaroo mother care pilot programmes were initially introduced at 21 health-care facilities, with 16 remaining functional. Health-care providers were trained by a pool of physician trainers to provide kangaroo mother care services; record-keeping and reporting systems were established; and managers and community health workers were prepared. Each service centre provided counselling to mothers on kangaroo mother care, ensured adequate nutrition through counselling about breastfeeding, provided support services to other centres and followed up mothers and babies (Fig. 1). The programme included four planned follow-up visits after discharge at weekly intervals. All eligible babies were managed according to standard facility protocols along with kangaroo mother care services. The details of the training and implementation have been described previously.^9^

The online kangaroo mother care data set was also launched in 2016. Data are entered by trained personnel within the facilities and periodically monitored by the Directorate General of Health Services for completeness and accuracy. Staff from the national newborn health programme evaluated kangaroo mother care data every month and acted on underreporting facilities. Kangaroo mother care services were monitored quarterly by government personnel through the national trainer pool, whereby professionals and programme personnel visited facilities and conducted onsite monitoring. This reference group provided expert opinions to service providers facing difficulties in implementing kangaroo mother care. Progressively, kangaroo mother care was introduced formally into the curricula of medical and nursing professionals. In parallel, various professional organizations of paediatricians, obstetricians, neonatologists and nursing colleagues endorsed the universal practice of kangaroo mother care.^9^

### Evaluation

We used quantitative methods to evaluate the implementation of the kangaroo mother care programme from January 2016 to March 2020. Our primary data source was the national data set for kangaroo mother care within the DHIS2 health management data platform (Health Information Systems Programme, Oslo, Norway). The data set includes both operational and outcome variables such as number of facilities having dedicated kangaroo mother care services, number of trained personnel, number of live births, number of babies with birth weight below 2000 g receiving the care, outcomes of babies who received the care, number of deaths, and follow-up rates of mother–baby pairs receiving the care. 

We used four outcome measures: (i) number of eligible babies enrolled in kangaroo mother care services; (ii) outcomes of babies receiving kangaroo mother care; (iii) mortality among babies receiving kangaroo mother care; and (iv) post-discharge follow-up rates. We included all clinically stable liveborn babies with birth weight below 2000 g at designated kangaroo mother care facilities. A clinically stable baby was a baby with normal respiratory and heart rate, normal oxygen saturation and without major congenital malformations. We report descriptive statistics with frequencies and percentages plotted using Excel version 2013 (Microsoft Corp. Redmond, United States of America). We also monitored the percentage of babies receiving kangaroo mother care who were: discharged as per the protocol, discharged on request, discontinued kangaroo mother care or died. We calculated mortality as the percentage of babies receiving kangaroo mother care who died within the facility while receiving kangaroo mother care. We calculated follow-up rates as the percentage of babies receiving each of the four planned weekly follow-up visits after discharge. 

Approval was taken from the Directorate General of Health Services for analysis of data from the database. No individual health records were accessed or reviewed.

## Results

The number of functioning kangaroo mother care corners was increasing over the study period, from 16 in 2016 to 108 in 2020. Among them 29 (26.9%) corners were in urban areas and the remaining 79 (73.1%) were in rural areas. The location of facilities was as follows: 79 (73.1%) in subdistrict health complexes, 21 (19.4%) in district hospitals and eight (7.4%) in medical college hospitals. 

Multiple development partners provided technical assistance to the government personnel at those sites. A total of 1196 health-care providers were trained from 2018–2020 in 60 batches, among whom 828 (69.2%) were nurses, 228 (19.1%) were midwives, and 140 (11.7%) were doctors. 

**Fig. 2:**
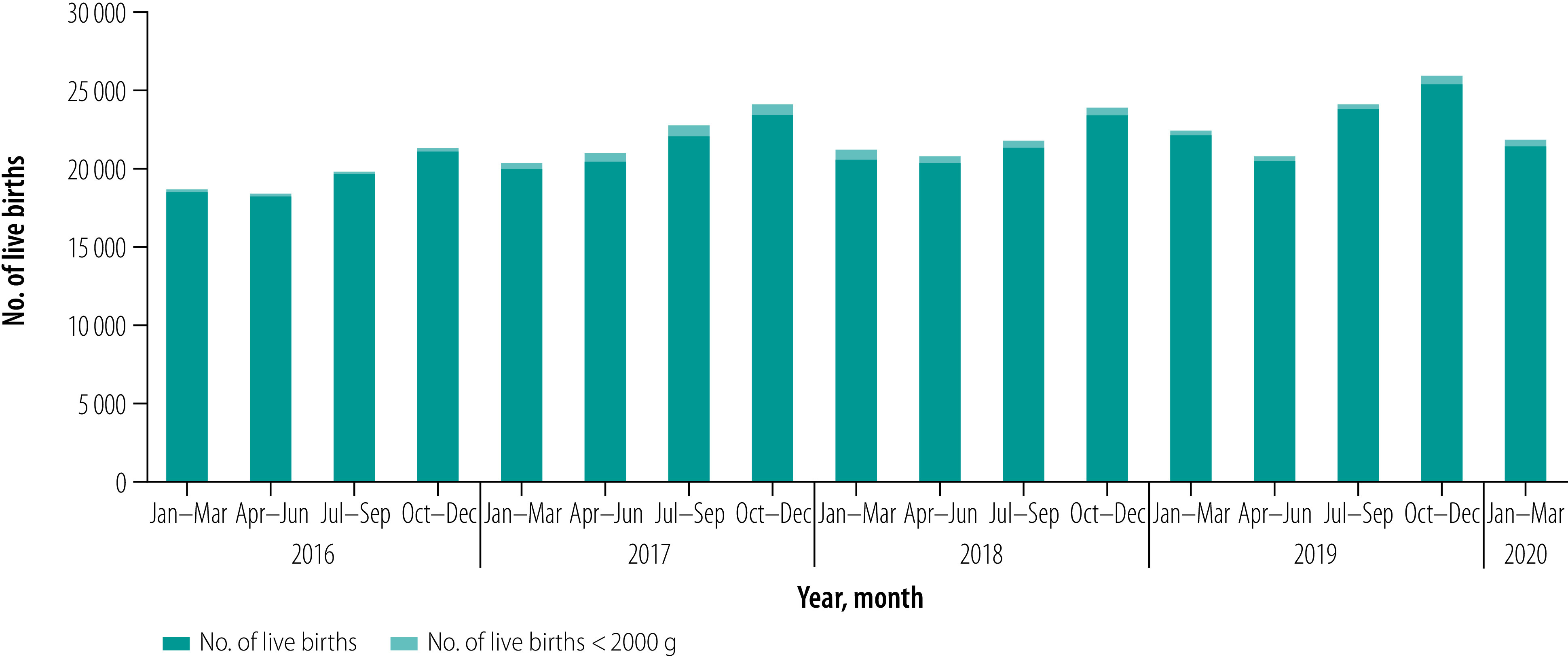
Total live births and babies born weighing less than 2000 g in designated kangaroo mother care centres, Bangladesh, January 2016 to March 2020

Source: We extracted quarterly data from the government’s DHIS2 health management data platform.

A total of 3 615 453 babies were born in the kangaroo mother care facilities during the study period, of whom 64 426 (1.8%) had birth weight less than 2000 g (Fig. 2). Table 1 shows the quarterly data set for the study duration.

**Table 1:** Quarterly data from designated kangaroo mother care centres, Bangladesh, January 2016 to March 2020

Variable	2016					2017					2018					2019					2020
	Jan–Mar	Apr–Jun	Jul–Sep	Oct–Dec		Jan–Mar	Apr–Jun	Jul–Sep	Oct–Dec		Jan–Mar	Apr–Jun	Jul–Sep	Oct–Dec		Jan–Mar	Apr–Jun	Jul–Sep	Oct–Dec		Jan–Mar
No. of live births	184 507	180 868	196 985	210 587		199 487	204 758	221 168	234 509		204 851	202 673	213 159	234 592		220 182	204 014	236 430	253 208		213 475
No. of babies < 2000 g	792	847	754	1 061		3 400	3 792	5 590	6 491		5 430	4 459	4 286	4 948		3 964	4 181	4 763	5 442		4 226
No. of babies < 2000 g starting kangaroo mother care	37	30	54	73		137	53	74	144		268	332	345	596		771	688	947	944		917
% of babies < 2000 starting kangaroo mother care	4.7	3.5	7.2	6.9		4.0	1.4	1.3	2.2		4.9	7.5	8.1	12.1		19.5	16.5	19.9	17.4		21.7
**Place of birth before kangaroo mother care, no. of babies**																					
Born in the facility	22	17	28	33		20	18	31	48		106	170	174	349		386	315	454	506		497
Born outside the facility	15	13	26	40		117	35	43	96		162	162	171	247		385	373	493	438		420
**Outcomes of kangaroo mother care, no. of babies**																					
Discharged as per protocol	15	12	11	21		26	18	32	56		123	161	183	339		401	277	523	569		495
Discharged on request	9	12	27	25		15	16	23	59		106	110	126	224		207	187	240	264		316
Referred or discontinued due to complications	4	6	17	17		12	16	22	32		28	30	35	41		52	27	61	95		79
Died	0	2	0	1		2	1	2	1		4	3	2	20		3	2	14	11		9
**Follow-up after kangaroo mother care, no. of babies**																					
Received 1st follow-up	14	18	21	17		17	35	33	54		89	137	187	222		346	267	419	406		436
Received 2nd follow-up	0	0	0	0		7	20	11	20		55	56	70	105		233	167	268	279		317
Received 3rd follow-up	0	0	0	0		4	15	7	13		33	34	38	55		141	117	132	191		201
Received 4th follow-up	0	0	0	0		8	9	7	12		20	12	26	35		101	60	93	126		113

Source: We extracted data from the government’s DHIS2 health management data platform.

### Enrolment

**Fig. 3:**
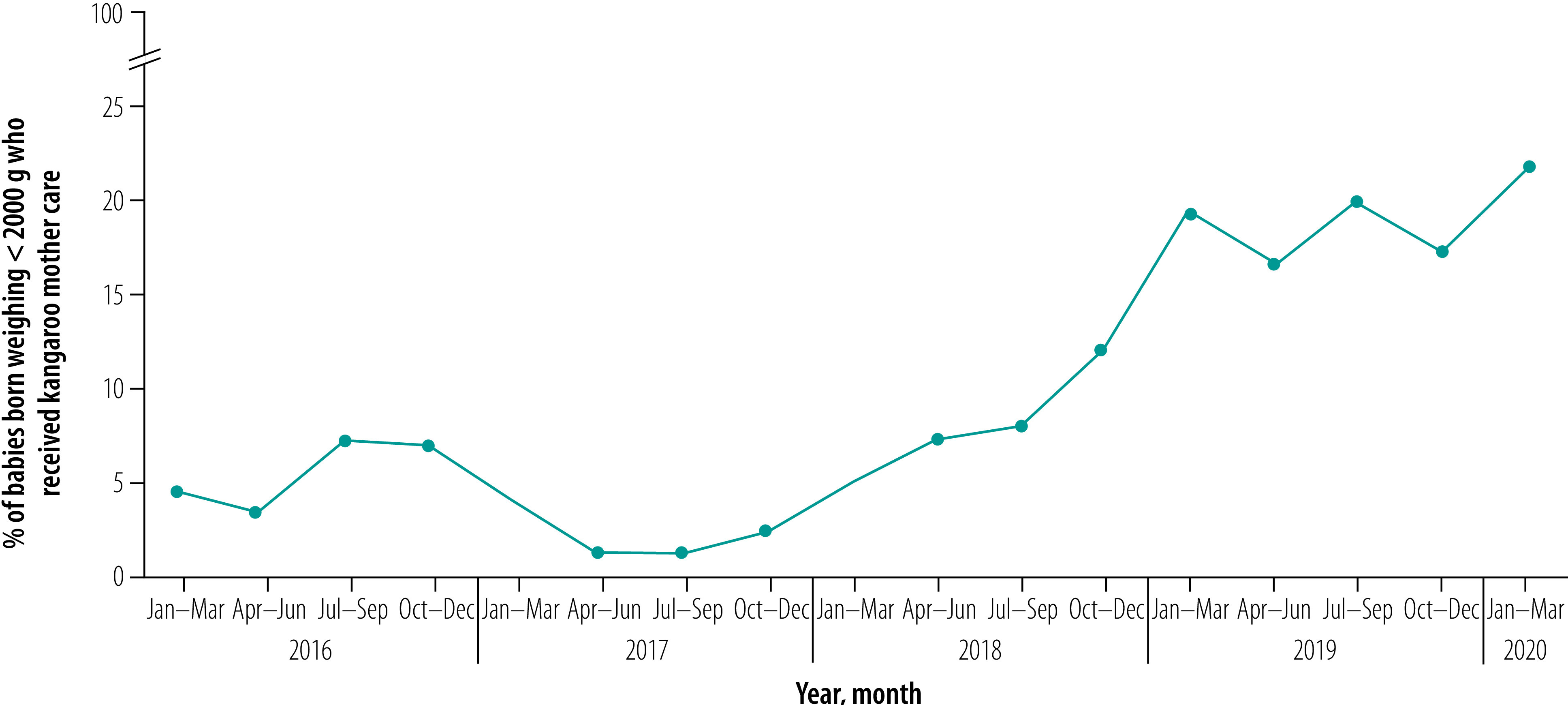
Percentage of eligible babies who received kangaroo mother care, Bangladesh, January 2016 to March 2020

Note: We included all clinically stable liveborn babies with birth weight below 2000 g at designated kangaroo mother care facilities. A clinically stable baby was a baby with normal respiratory and heart rate, normal oxygen saturation and without major congenital malformations.

Source: We extracted quarterly data from the government’s DHIS2 health management data platform.

Fig. 3 shows the enrolment of babies to kangaroo mother care. Over the period January 2016 to March 2020,  6410 babies with birth weight under 2000 g received kangaroo mother care in the facilities. In the first quarter of the implementation 4.7% (37/792) of eligible babies received kangaroo mother care. However, the percentage decreased to 1.4% (53/3792) in April to June 2017, despite the higher number of eligible babies identified. We can attribute the decrease to multiple factors such as poor compliance, lack of familiarity with protocols and missing cases. From April 2018, after implementation of corrective measures, such as more stringent continuous monitoring and training of additional providers, the situation improved and in the last quarter of the study 21.7% (917/4226) of eligible babies received kangaroo mother care. 

### Outcomes

**Fig. 4:**
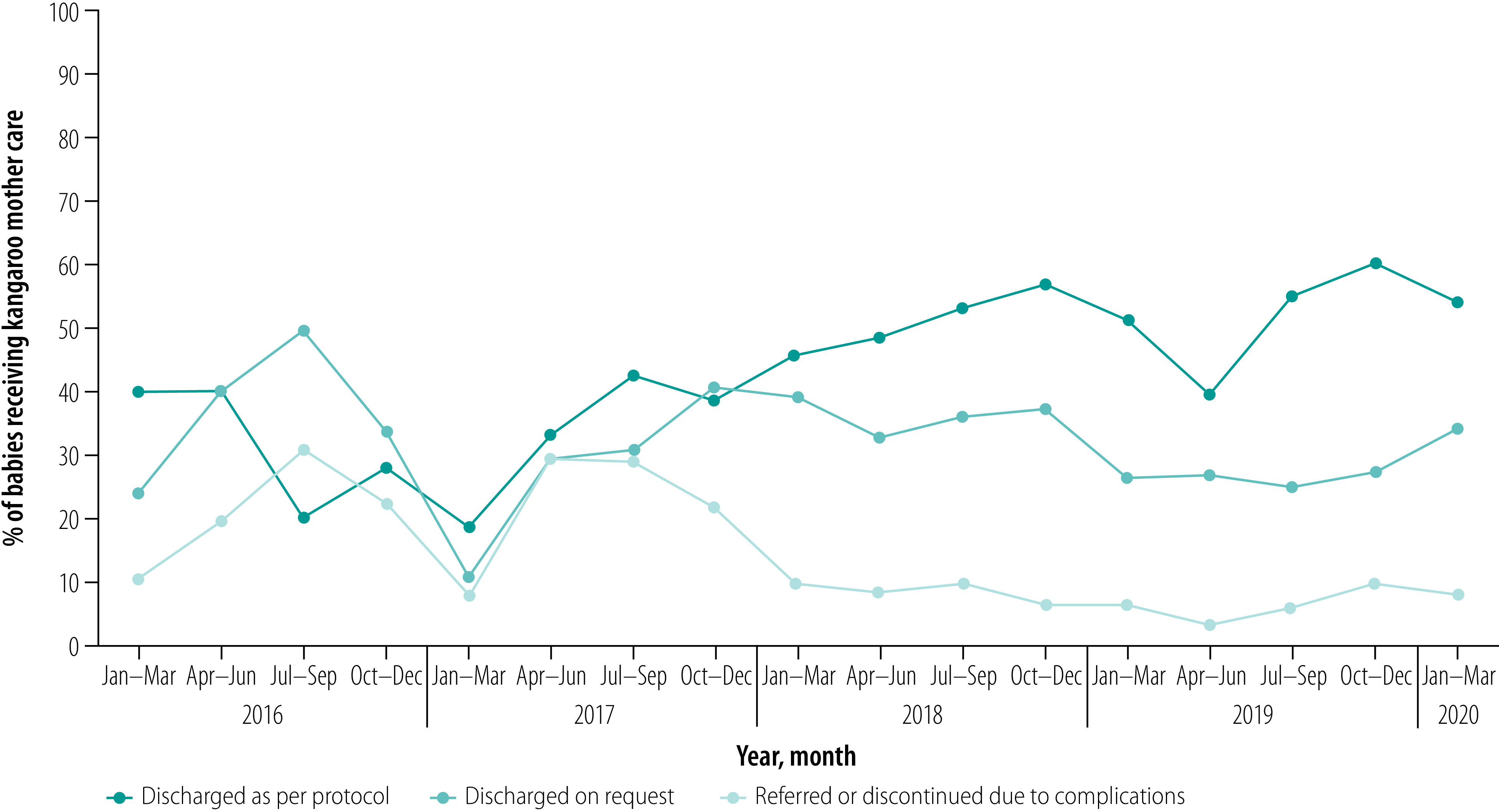
Outcomes of babies who received kangaroo mother care, Bangladesh, January 2016 to March 2020

Source: We extracted data from the government’s DHIS2 health management data platform.

Fig. 4 shows the trend in outcomes of babies receiving kangaroo mother care. The proportion of eligible babies receiving the care and discharged as per the protocol showed an upward trend, reaching 50–60% in the last three quarters. The remaining 40–50% of babies were discharged at a later period after resolution of problems such as difficulty establishing breastfeeding or supplemental feeding, hypothermia and electrolyte disturbances. The proportion of babies who received kangaroo mother care but later discontinued care or were referred to higher-level facilities such as special care newborn units or neonatal intensive care units continued to drop from 30% during April–September 2017 to below 10% during the last eight quarters of the study period.

**Fig. 5:**
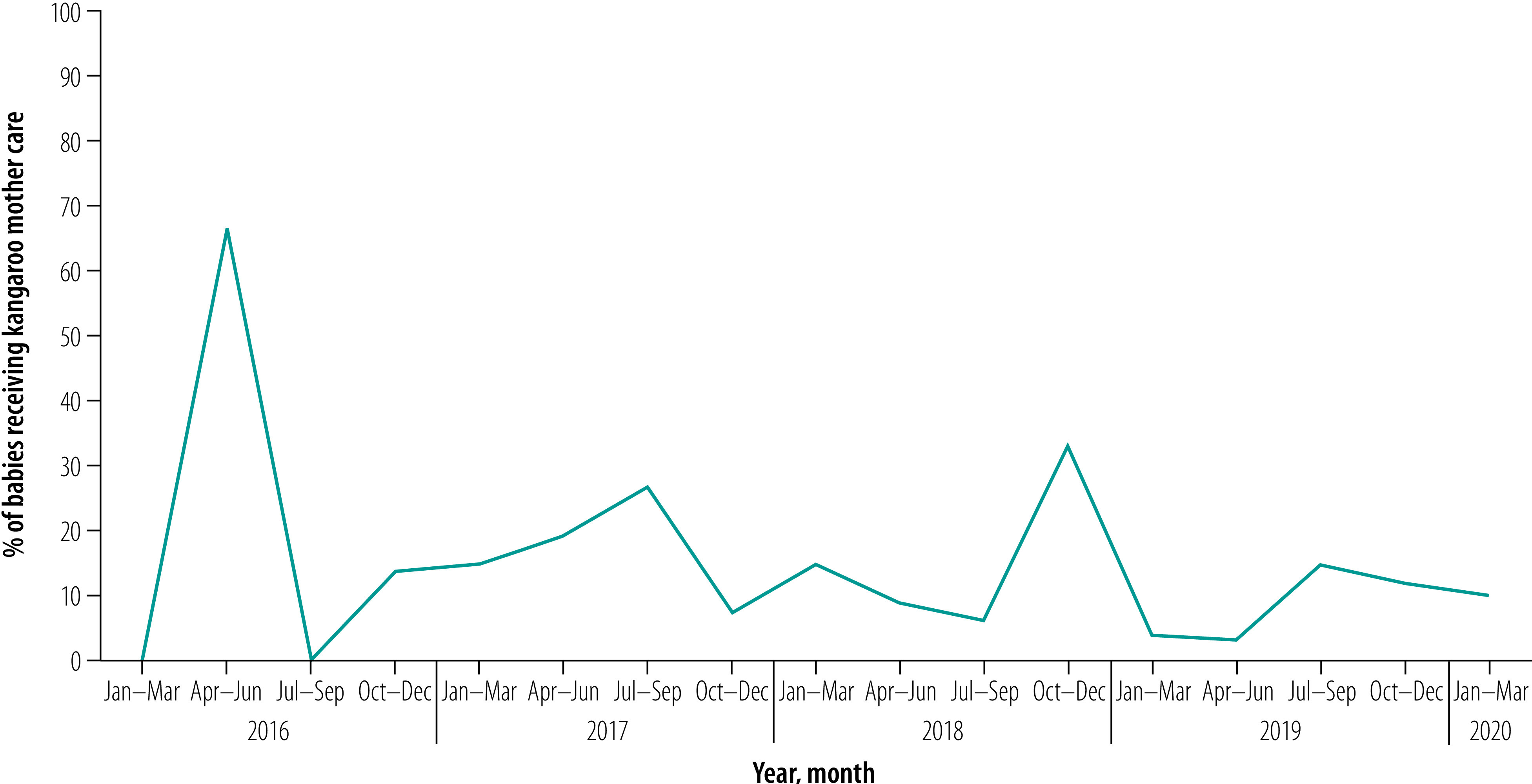
Deaths of babies who received kangaroo mother care, Bangladesh, January 2016 to March 2020

Note: We calculated the percentage of babies dying within the facility among babies receiving kangaroo mother care.

Source: We extracted quarterly data from the government’s DHIS2 health management data platform.

The overall mortality among the babies receiving kangaroo mother care was 1.2% (77/6410), with a downward trend over the study period (Fig. 5). The data showed large quarterly fluctuations in mortality, sometimes due to lower numbers of deaths and sometimes due to more babies receiving kangaroo mother care during the first few quarters of the programme. Fewer fluctuations were found during the later part of the study as the number of enrolments increased. 

### Follow-up

**Fig. 6:**
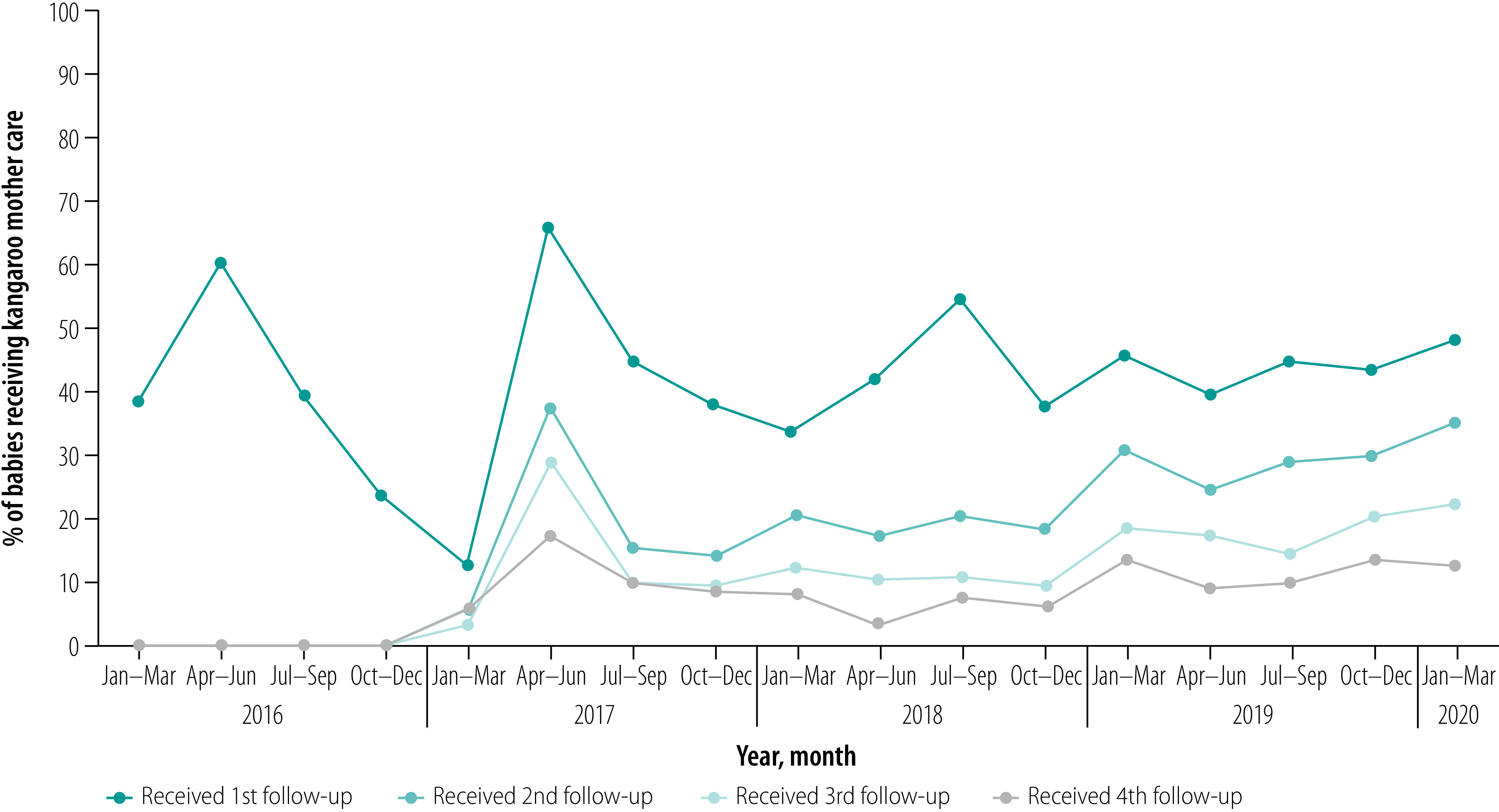
Follow-up of babies who received kangaroo mother care, Bangladesh, January 2016 to March 2020

Source: We extracted quarterly data from the government’s DHIS2 health management data platform.

Fig. 6 shows the follow-up rates for babies who received kangaroo mother care. Only the first of the four follow-up visits was sustained with 30–50% of babies receiving one follow-up visit until the end of the evaluation period. The percentage receiving second, third and fourth follow-up visits was suboptimal, although it improved from a baseline of 0% to around 15–20% in the last quarters of the study period.

## Discussion

We report the implementation and initial evaluation of the kangaroo mother care programme in Bangladesh. We noticed a decreasing trend in mortality among babies with birth weight below 2000 g receiving kangaroo mother care in the designated facilities, with an overall mortality of 1.2% over the study period. This value is significantly lower than the historical neonatal mortality (deaths before or at 28 days of life) of 19% among babies born weighing less than 2500 g in Bangladesh.^14^ Of note, the kangaroo mother care programme enrolled babies with birth weight less than 2000 g – a more vulnerable group that is expected to have higher mortality than babies weighing between 2000 g and 2500 g. However, we counted deaths that occurred while in the kangaroo mother care facilities and not neonatal deaths after discharge from the facilities. Other parameters showed a modest improvement. For example, enrolment in kangaroo mother care, although improved, reached 20%. Follow-up after discharge – a key element of the WHO kangaroo mother care guidelines – was low, especially for the second, third and fourth follow-up visits.

In an earlier randomized controlled trial of community-based kangaroo mother care among babies of all birth weights in Bangladesh, no overall difference in mortality was found although there was a reduction in mortality among babies with birth weight less than or equal to 2000 g.^15^ Among newborns randomized to community kangaroo mother care, 77.4% (1565/2022) of infants received any skin-to-skin care, with only 23.8% of 1985 infants receiving skin-to-skin care for more than 7 hours per day in the first 2 days of life. Similarly, a large randomized controlled trial studying community initiated kangaroo mother care in rural India reported a 30% increase in neonatal survival (hazard ratio: 0.7; 95% confidence interval, CI: 0.51–0.96) and a 25% increase in infant survival at 6 months (hazard ratio: 0.75; 95% CI: 0.60–0.93).^16^ We add to the literature on kangaroo mother care and demonstrate that kangaroo mother care, implemented in low-resource settings at national level, can also result in a progressive reduction of mortality among neonates.

Our study has some limitations. The data were incomplete, especially during the initial days of the study, due to logistical challenges of collecting and monitoring the data. Even in the best performing quarter, about four fifths of eligible babies did not receive kangaroo mother care. We did not evaluate the factors that could have been associated with improved survival (such as duration and number of days receiving kangaroo mother care sessions, breastfeeding, degree of prematurity and birth weight). We did not have a control group, and we could only compare our data with historical norms. Kangaroo mother care facilities were established across different geographical locations; however, there was no stratification or sampling to ensure systematic representation of Bangladesh’s population. 

Other factors could also limit applicability of the findings to other countries. Although there is a WHO standardized definition of kangaroo mother care,^10^ the practice and implementation of the components of kangaroo mother care (skin-to-skin care, exclusive breastfeeding, early discharge home with appropriate follow-up and monitoring) vary widely.^17^ The programme was implemented in Bangladesh with support from international and national partners with a long history of working in public health sectors. There were other contextual variables such as support from the government, involvement of health professionals and community engagement that may not be replicated in other countries.

We recognize that there are several areas of the programme that can be improved. Nonfunctioning kangaroo mother care facilities can become functioning through supportive measures such as direct visits to facilities, identifying problems and monitoring and supervision. More eligible babies should receive kangaroo mother care. In Bangladesh around 50% of babies are delivered at home and only 53% are attended by medical personnel.^14^ We believe there is an opportunity to initiate kangaroo mother care at home through preparation of mothers during the antenatal period and home visits by trained health-care professionals. As the kangaroo mother care programme expands, private hospitals, where a significant number of babies are born, should be included in the programme. Further capacity-building and training will provide adequate manpower to keep kangaroo mother care facilities running in case of staff resignations or relocation. Incentives can be provided to the better-performing kangaroo mother care facilities and events can be organized to showcase innovations within facilities. We also noted poor post-discharge follow-up visits, especially the second, third and fourth follow-ups. Probable factors contributing to poor follow-up were loss of contact with mothers, lack of appropriate counselling to mothers and low awareness of the programme among recipients. We have been rolling out mitigation measures progressively such as increasing the number of trained personnel, increasing community involvement with social and behavioural change communication, procurement of essential commodities for the service, and education to further improve post-discharge follow-up. Finally, we intend to expand our database to include relevant variables for further research and more effective evaluation. A separate data set can be created for babies born in the community and in private hospitals. 

We were able to develop a set of recommendations for scaling up and strengthening of the programme (Box 1). We have learnt that strong commitment from the government and development partners and a positive working environment are important for a kangaroo mother care programme to run successfully in low-resource settings. However, such programmes must have continuous monitoring, adjustments and improvements. For example, at the initial phase of our programme, the number of babies receiving kangaroo mother care was low. The enrolment reached a low of just over 1% soon after the programme started. We explored factors contributing to low enrolment through informal surveys among the service providers. We determined lack of knowledge about kangaroo mother care resulting in lack of motivation from the community and service providers and limited numbers of trained staff. As a direct consequence of these factors there were many nonfunctional kangaroo mother care corners that were major contributors to poor enrolment. Encouragingly, stringent continuous monitoring helped us to detect this trend and allowed us to implement corrective measures. These measures included training additional health-care providers and implementing internal and external supervision of kangaroo mother care activities, with resultant improvement from April 2018. The Directorate General of Health Services and Directorate General of Family Planning, both under the health ministry, plan to train an additional 41 700 and 20 820 health professionals on kangaroo mother care, respectively, for the period 2020–2022. In addition, the Directorate General of Health Services is planning to expand kangaroo mother care corners in another 125 facilities by 2020–2021, covering all district- and subdistrict-level health facilities by 2022. Our preliminary data provide useful lessons for implementation of kangaroo mother care services at the national level

Box 1 **Lessons learnt for implementation of kangaroo mother care services**

**Monitoring poor performance**

Nonfunctional kangaroo mother care centres need to be identified and transformed into functional centres. Improvements can be made by direct visits to health-care facilities, identifying their problems and supportive monitoring and supervision.

**Capacity-building**

An adequate number of health-care providers providing kangaroo mother care service from a single centre need to be trained so that the programme can continue in case of high turnover of trained staff, especially nurses.

**Empowering midwives and community health-care providers**

In Bangladesh, around half of deliveries that take place at home are followed up by community health-care providers.^14^ On the other hand, normal deliveries at government hospitals are mainly supported by midwives. Empowering these two groups with kangaroo mother care services can improve programme outcomes.

**Preventing prematurity and low birth weight**

The kangaroo mother care programme should run in conjunction with antenatal services to prevent premature birth and low birth weight through interventions such as nutritional management, frequent antenatal follow-up and health education.

**Expanding the data set**

We intend to collect more variables on kangaroo mother care (such as duration of kangaroo mother care, duration of each session, incidence of hypothermia and infection, percentage of babies requiring oxygen, and babies on nasogastric or orogastric tube feeding during kangaroo mother care). A separate data set can be created for babies born in the community and private hospitals.

**Case tracking**

Most of the mothers who undertook kangaroo mother care in Bangladesh were lost to follow-up. Lack of motivation and limitations in contacting parents were the major challenges. Therefore, family participation during kangaroo mother care counselling and using a designated mobile phone in the hospital for tracing mothers could be helpful.

**Recognizing staff performance**

Kangaroo mother care events could be arranged in communities so that facilities can share information about their care practices and outcomes and can be rewarded for good performance. The best performing nurse or midwife from each facility could be given a public recognition award to acknowledge their contributions and motivate others.

**Boosting research**

Research on kangaroo mother care should be undertaken in different hospitals to determine the best ways to overcome challenges. Similarly, existing data sets such as DHIS2 can also be used for future large-scale research.

## References

[1] Born too soon: the global action report on preterm birth. Geneva: World Health Organization; 2012. Available from: https://www.who.int/reproductivehealth/publications/maternal_perinatal_health/9789241503433/en/ [cited 2021 Aug 29].

[2] WHA global nutrition targets 2025: low birth weight policy brief. Geneva: World Health Organization; 2014. Available from: https://apps.who.int/nutrition/publications/globaltargets2025_policybrief_lbw/en/index.html [cited 2021 Aug 29].

[3] Lawn JE, Davidge R, Paul VK, von Xylander S, de Graft Johnson J, Costello A, et al. Born too soon: care for the preterm baby. Reprod Health. 2013;10(S1) Suppl 1:S5. 10.1186/1742-4755-10-S1-S5 24625233 PMC3828583

[4] Engmann C, Wall S, Darmstadt G, Valsangkar B, Claeson M; participants of the Istanbul KMC Acceleration Meeting. Consensus on kangaroo mother care acceleration. Lancet. 2013 Nov 30;382(9907):e26–7. 10.1016/S0140-6736(13)62293-X 24246562

[5] Bergh AM, Charpak N, Ezeonodo A, Udani RH, Rooyen EV. Education and training in the implementation of kangaroo mother care. SAJCH. 2012;6(2):38–45.

[6] Blencowe H, Cousens S, Oestergaard MZ, Chou D, Moller AB, Narwal R, et al. National, regional, and worldwide estimates of preterm birth rates in the year 2010 with time trends since 1990 for selected countries: a systematic analysis and implications. Lancet. 2012 Jun 9;379(9832):2162–72. 10.1016/S0140-6736(12)60820-4 22682464

[7] Liu L, Oza S, Hogan D, Perin J, Rudan I, Lawn JE, et al. Global, regional, and national causes of child mortality in 2000-13, with projections to inform post-2015 priorities: an updated systematic analysis. Lancet. 2015 Jan 31;385(9966):430–40. 10.1016/S0140-6736(14)61698-6 25280870

[8] Bangladesh Demographic and Health Survey 2014 – key indicators. Dhaka: Director General of Health Services; 2014. Available from: https://dghs.gov.bd/images/docs/Other_Publication/BanladeshDemographicHealthSurvey2014.pdf [cited 2021 Aug 29].

[9] Kangaroo mother care in Bangladesh. Saving newborn lives. London: Save The Children; 2018. Available from: https://www.mcsprogram.org/wp-content/uploads/2018/06/Bangladesh-KAP-Summary-Sheet.pdf [cited 2021 Aug 18].

[10] Kangaroo mother care: a practical guide. Geneva: World Health Organization; 2003. Available from: https://www.who.int/publications/i/item/9241590351 [cited 2020 Aug 29].

[11] Every newborn action plan. Country progress tracking report. Geneva: United Nations Children’s Fund; 2015. Available from: https://data.unicef.org/wp-content/uploads/2016/11/160511-ENAP-country-progress-tracking-report-2015-1.pdf [cited 2020 Aug 29].

[12] The 4th Health, Population and Nutrition Sector Program, Maternal, Neonatal, Child and Adolescent Health Operation Plan, January 2017–June 2022. Dhaka: Ministry of Health and Family Welfare; 2017. Available from: https://dghs.gov.bd/images/docs/OP/2018/MNC_AH.pdf [cited 2021 Aug 29].

[13] Rubayet S, Shahidullah M, Hossain A, Corbett E, Moran AC, Mannan I, et al.; Bangladesh Newborn Change and Future Analysis Group. Newborn survival in Bangladesh: a decade of change and future implications. Health Policy Plan. 2012 Jul;27(3) Suppl 3:iii40–56. 10.1093/heapol/czs044 22692415

[14] Bangladesh Demographic and Health Survey 2017–18. Dhaka and Rockville: National Institute of Population Research and Training and ICF; 2020. Available from: https://dhsprogram.com/pubs/pdf/FR344/FR344.pdf [cited 2021 Aug 29].

[15] Sloan NL, Ahmed S, Mitra SN, Choudhury N, Chowdhury M, Rob U, et al. Community-based kangaroo mother care to prevent neonatal and infant mortality: a randomized, controlled cluster trial. Pediatrics. 2008 May;121(5):e1047–59. 10.1542/peds.2007-0076 18450847

[16] Mazumder S, Taneja S, Dube B, Bhatia K, Ghosh R, Shekhar M, et al. Effect of community-initiated kangaroo mother care on survival of infants with low birthweight: a randomised controlled trial. Lancet. 2019 Nov 9;394(10210):1724–36. 10.1016/S0140-6736(19)32223-8 31590989

[17] Chan GJ, Labar AS, Wall S, Atun R. Kangaroo mother care: a systematic review of barriers and enablers. Bull World Health Organ. 2016 Feb 1;94(2):130–141J. 10.2471/BLT.15.157818 26908962 PMC4750435

